# London's New Club

**Published:** 1920-11-13

**Authors:** 


### London's New Club.

Sir Arthur Stanley was privileged to make an announcement at the extraordinary meeting of the College of Nursing last week, which sent a new thrill of satisfaction through the crowded rooms. This was no less than the most generous intention of Lord and Lady Cowdray to hand over the fine premises in Cavendish Square, which they have already presented to the members, fully furnished and equipped for the purposes of a residential club. The opening is foreshadowed for next June.

At the present moment when the cost of furnishing one small room may easily run to a hundred pounds, the munificence of the gift warms the imagination. And it is not merely the money that counts, though that is a considerable asset both in principal and interest. It is the lively personal interest dictating the whole benefaction which moves nurses to gratitude and enthusiasm. When the Club is ready for their occupation they will receive it as a mark of genuine sympathy with nurses' aims, of the appreciation of the difficulties nurses experience and the sacrifices they make for the relief of suffering, and, moreover, a special indication of interest in the ways adopted by the College of Nursing to improve the status of their profession.

It is abundantly clear that the furniture and equipment so cordially bestowed will be very different from what the College, even provided with sufficient funds, would have ventured in these hard times to get together. That is one of the charms of a "Gift-House." It may go beyond the sternly necessary. It may take account of beauty as well as of economy and durability. It can afford to be individual. It is spontaneous, and with all due deference to the taste of committees there is something far more distinctive about the personal choice of a well-inspired friend than about articles provided by collective prudence. The many hours of practical and laborious selection which the house will cost Lady Cowdray will bear fruit in happy hours for members of the College for generations to come, and in her devotion to their interests she builds a beautiful memorial of her own devotion to the sick.

